# Norepinephrine to increase blood pressure in endotoxaemic pigs is associated with improved hepatic mitochondrial respiration

**DOI:** 10.1186/cc6956

**Published:** 2008-07-14

**Authors:** Tomas Regueira, Bertram Bänziger, Siamak Djafarzadeh, Sebastian Brandt, Jose Gorrasi, Jukka Takala, Philipp M Lepper, Stephan M Jakob

**Affiliations:** 1 Department of Intensive Care Medicine, Bern University Hospital (Inselspital) and University of Bern, Freiburgstrasse, CH-3010 Bern, Switzerland; 2 Department of Anesthesiology and Pain Therapy, Bern University Hospital (Inselspital) and University of Bern, Freiburgstrasse, CH-3010 Bern, Switzerland

## Abstract

**Introduction:**

Low blood pressure, inadequate tissue oxygen delivery and mitochondrial dysfunction have all been implicated in the development of sepsis-induced organ failure. This study evaluated the effect on liver mitochondrial function of using norepinephrine to increase blood pressure in experimental sepsis.

**Methods:**

Thirteen anaesthetized pigs received endotoxin (*Escherichia coli *lipopolysaccharide B0111:B4; 0.4 μg/kg per hour) and were subsequently randomly assigned to norepinephrine treatment or placebo for 10 hours. Norepinephrine dose was adjusted at 2-hour intervals to achieve 15 mmHg increases in mean arterial blood pressure up to 95 mmHg. Systemic (thermodilution) and hepatosplanchnic (ultrasound Doppler) blood flow were measured at each step. At the end of the experiment, hepatic mitochondrial oxygen consumption (high-resolution respirometry) and citrate synthase activity (spectrophotometry) were assessed.

**Results:**

Mean arterial pressure (mmHg) increased only in norepinephrine-treated animals (from 73 [median; range 69 to 81] to 63 [60 to 68] in controls [*P *= 0.09] and from 83 [69 to 93] to 96 [86 to 108] in norepinephrine-treated animals [*P *= 0.019]). Cardiac index and systemic oxygen delivery (DO_2_) increased in both groups, but significantly more in the norepinephrine group (*P *< 0.03 for both). Cardiac index (ml/min per·kg) increased from 99 (range: 72 to 112) to 117 (110 to 232) in controls (*P *= 0.002), and from 107 (84 to 132) to 161 (147 to 340) in norepinephrine-treated animals (*P *= 0.001). DO_2 _(ml/min per·kg) increased from 13 (range: 11 to 15) to 16 (15 to 24) in controls (*P *= 0.028), and from 16 (12 to 19) to 29 (25 to 52) in norepinephrine-treated animals (*P *= 0.018). Systemic oxygen consumption (systemic VO_2_) increased in both groups (*P *< 0.05), whereas hepatosplanchnic flows, DO_2 _and VO_2 _remained stable. The hepatic lactate extraction ratio decreased in both groups (*P *= 0.05). Liver mitochondria complex I-dependent and II-dependent respiratory control ratios were increased in the norepinephrine group (complex I: 3.5 [range: 2.1 to 5.7] in controls versus 5.8 [4.8 to 6.4] in norepinephrine-treated animals [*P *= 0.015]; complex II: 3.1 [2.3 to 3.8] in controls versus 3.7 [3.3 to 4.6] in norepinephrine-treated animals [*P *= 0.09]). No differences were observed in citrate synthase activity.

**Conclusion:**

Norepinephrine treatment during endotoxaemia does not increase hepatosplanchnic flow, oxygen delivery or consumption, and does not improve the hepatic lactate extraction ratio. However, norepinephrine increases the liver mitochondria complex I-dependent and II-dependent respiratory control ratios. This effect was probably mediated by a direct effect of norepinephrine on liver cells.

## Introduction

Septic shock is associated with high mortality, especially when antibiotic treatment and fluid resuscitation are delayed and oxygen delivery remains insufficient [1]. Despite recent recommendations on blood pressure targets in septic shock [2,3], the goals in clinical trials vary substantially [4]. Recently, Varpula and coworkers [1] showed that mean arterial pressure (MAP) is the most powerful predictor of mortality in septic shock, emphasizing the importance of global perfusion pressure for survival. Also, according to a recent systematic review of randomized clinical trials that used MAP as the goal of resuscitation in septic patients [4], the minimum and maximum targets for MAP ranged from 60 to 100 mmHg [5-13]. The authors of the review concluded that there was wide variation in the goals chosen for the studies and that this variation may lead to bias in the interpretation of study results. More intriguingly, the achieved blood pressure in these trials was substantially higher than the target blood pressure.

Vasoactive drugs such as norepinephrine (noradrenaline) are commonly used to maintain a certain MAP level in septic (shock) patients. However, the regional haemodynamic and metabolic effects of norepinephrine during sepsis are not fully understood and are controversial [14-19]. Systemically, norepinephrine increases cardiac output and oxygen delivery and consumption [18], and at the regional level it has been reported to increase portal vein flow [20], total splanchnic blood flow and oxygen delivery during sepsis [14].

Other studies have identified unchanged mesenteric flows [19], total splanchnic blood flow and oxygen uptake [17-19]. In a crossover study, Guerin and colleagues [16] compared norepinephrine with dopamine in septic patients, with the MAP goal being 80 mmHg. They reported that the drugs were associated with similar splanchnic blood flow and hepatic oxygen consumption, but that patients treated with norepinephrine exhibited higher levels of hepatic lactate uptake and lower values of the lactate-pyruvate ratio, suggesting improved hepatic energy balance with norepinephrine. Also, Revelly and coworkers [21] reported that, during distributive shock induced by endotoxaemia in pigs, norepinephrine prevented the decrease in intestinal mucosal ATP content, which was observed only in fluid-resuscitated animals. This also suggests improved energy balance with norepinephrine. Only one study evaluated the effects of increasing MAP from 65 to 85 mmHg using norepinephrine [22]. In this study, conducted in 10 patients with septic shock, increasing MAP with norepinephrine was associated with a significant increase in cardiac index, left ventricular stroke work index, heart rate and systemic oxygen delivery. Because systemic oxygen consumption, lactate concentrations, capillary blood flow, urine output and gastric mucosal partial carbon dioxide tension were not altered, the benefit of increasing blood pressure was questioned.

Growing evidence suggests that mitochondrial damage and dysfunction play an important role in the pathogenesis of sepsis-induced organ failure [23,24]. Mitochondrial dysfunction can be characterized by inability of the mitochondria to couple oxygen consumption with energy production completely. In a long-term, fluid-resuscitated, faecal peritonitis model in mice, Breadley and colleagues [25] demonstrated that severity of and mortality from sepsis were related to nitric oxide over-production and consequent complex I inhibition and ATP depletion.

Because norepinephrine affects at least systemic haemodynamics, it may also affect liver mitochondrial function, either through an indirect regional haemodynamic mechanism (for instance, an increase in perfusion pressure/flow relationship or an increase in regional oxygen delivery) or through a direct cellular mechanism. A direct cellular effect of norepinephrine on isolated hepatocytes has been described and includes stimulation of α-adrenergic receptor, increasing cytosol and intra-mitochondrial calcium levels, and activation of dehydrogenases of the citrate cycle [26,27]. Therefore, in the present study we tested whether a stepwise increase in blood pressure with norepinephrine, in the setting of a clinically relevant range of pressures between 60 and 95 mmHg in a model of endotoxic sepsis, is associated with a beneficial effect on liver blood flow, oxygen delivery and consumption, and mitochondrial function.

## Materials and methods

The study was performed in accordance with the National Institutes of Health guidelines for the care and use of experimental animals and with the approval of the Animal Care Committee of the Canton of Bern, Switzerland.

### Animal preparation and experimental setting

Thirteen pigs (weight 37 to 44 kg) were fasted overnight and premedicated with ketamine (20 mg/kg) and xylazine (2 mg/kg intramuscularly), followed by intravenous administration of midazolam (0.5 mg/kg) and atropine (0.02 mg) for endotracheal intubation. The animals were ventilated with a volume-controlled ventilator (Servo ventilator 900 C; Siemens, Elema, Sweden) with 5 cmH_2_O end-expiratory pressure. The fraction of inspired oxygen and positive end-expiratory pressure were adjusted to keep arterial oxygen tension levels between 13.3 kPa (100 mmHg) and 20 kPa (150 mmHg), and the minute ventilation was adjusted to maintain arterial carbon dioxide tension levels between 4.5 and 5.5 kPa (34 to 41 mmHg). Anaesthesia was maintained with propofol (4 mg/kg per hour) and fentanyl (45 μg/kg per hour during surgery and 30 μg/kg per hour afterward). After canulation of jugular and femoral veins and the femoral artery for the placement of hepatic venous, and pulmonary arterial and femoral arterial catheters, a laparatomy was performed and a urinary bladder catheter was inserted. Afterward, ultrasound Doppler flow probes (Transonic^® ^System Inc., Ithaca, NY, USA), which had previously been calibrated *in vitro*, were positioned around the carotid and hepatic arteries and the portal vein. Finally, a catheter was inserted into the portal vein for blood sampling. After the procedure the abdominal wall was sewn closed.

Regional blood flows and portal vein pressure were monitored continuously during the experiment and stored in a computer (WinDaq^®^; DATAQ instruments, Akron, OH, USA). Electrocardiogram, heart rate, and carotid and pulmonary arterial and central venous pressures were monitored continuously, and pulmonary artery occlusion pressure intermittently. Cardiac output was assessed hourly with three measurements using the thermodilution technique (S/5 Compact Critical Care monitor; Datex-Ohmeda, Helsinki, Finland). Central temperature was recorded from the thermistor in the pulmonary artery catheter (cardiac output/mixed venous oxygen saturation catheter; Edwards Lifesciences, Munich, Germany) and peripheral temperature from the tip of a toe. All of these variables were recorded using an electronic patient data management system (Clinisoft, GE Healthcare, Helsinki, Finland). The system automatically stores median values in a database every 2 minutes.

Once the experiment was started, manipulation was avoided to minimize the possibility of flow probe displacement. At the end of the experiment, the correct position of each probe and catheter was controlled visually.

### Experimental protocol

After surgery, a 1-hour period was allowed for haemodynamic stabilization. After this period, pigs were randomized for a 10-hour experiment into two groups: endotoxin plus progressively increasing goals of MAP, achieved by increasing doses of norepinephrine (norepinephrine group; n = 7); or endotoxin alone (control group; n = 6). Initially, six pigs were randomized per group, but because one mitochondrial respiration experiment was missed in the norepinephrine group, one more pig was included in this group.

Endotoxin (*Escherichia coli *lipopolysaccharide B0111:B4, 20 mg/l in 5% dextrose; Difco Laboratories, Detroit, MI, USA) was infused into the right atrium in all animals. The initial infusion rate was 0.4 μg/kg per hour until the mean pulmonary arterial pressure reached 35 mmHg. The infusion was then stopped and subsequently adjusted to maintain moderate pulmonary artery hypertension (mean pulmonary artery pressure 25 to 30 mmHg). If systemic hypotension persisted below 50 mmHg, then the endotoxin infusion was temporarily stopped and 50 ml of hydroxyethyl starch (Voluven 6%; Fresenius, Stans, Switzerland) was administered. All pigs received Ringer's lactate solution 5 ml/kg per hour, and glucose 50% solution was administered in order to maintain a blood glucose concentration between 3.5 and 6 mmol/l.

In the norepinephrine group, norepinephrine was continuously infused to reach the following pre-defined MAP goals: from 0 to 2 hours there was no specific goal; from 2 to 4 hours the goal was 50 mmHg; from 4 to 6 hours the goal was 65 mmHg; from 6 to 8 hours the goal was 80 mmHg; and from 8 to 10 hours the goal was 95 mm Hg. The MAP goal for the control group was between 60 and 70 mmHg for the whole experiment. If the MAP of the control group was below 60 mmHg, then additional boluses of 50 to 100 ml of colloids (Voluven 6%; Fresenius) were given. If the MAP of the animals in the norepinephrine group was found to be above the target for the corresponding time point, then no norepinephrine was added.

### Blood sampling

Blood samples for the measurement of haemoglobin, lactate and blood gases were taken at baseline and every 2 hours afterward from pulmonary and femoral arteries and from portal and hepatic veins (ABL 520 and OSM 3 [pig module; Radiometer, Copenhagen, Denmark], and YSI 2300 Stat Plus [Yellow Springs Instruments, Yellow Springs, OH, USA]).

### Liver mitochondrial isolation

At the end of the experiment, liver tissue samples of approximately 15 g were taken from the living animal for isolation of mitochondria. Afterward, the animals were killed with an overdose of intravenous potassium chloride. Isolation of liver mitochondria was performed immediately at 4°C using a standard procedure based on differential centrifugation [28]. The liver samples were rapidly immersed in ice-cold isolation buffer (220 mmol/l mannitol, 70 mmol/l sucrose, 5 mmol/l morpholinopropane sulfonic acid [pH 7.4]), minced with scissors and homogenized in 10 ml of homogenization medium per gram of tissue (isolation buffer plus 2 mmol/l ethyleneglycol tetra-acetate) in a Potter Elvehjem homogenizer with a loose-fitting Teflon pestle. The homogenate was then centrifuged for 10 minutes at 700 *g*. The supernatant was collected and centrifuged again for 10 minutes at 7,000 *g*. The supernatant was discarded at this time; the pellet was then resuspended in isolation buffer and centrifuged twice for 10 minutes at 7,000 *g *for further purification of the mitochondria. The pellets were then suspended in buffer at a final concentration of 50 to 100 mg mitochondrial protein per milliliter.

### Determination of mitochondrial oxygen consumption by high-resolution respirometry

Protein concentration was determined spectrophotometrically with the Biuret method using bovine serum albumin as a standard. Respiratory rates were determined at a final mitochondrial protein concentration of 0.4 mg/ml. Respiration was measured at 37°C in 2 ml glass chambers using the High Resolution Oxygraph (OROBOROS; Oxygraph-2k, Graz, Austria). The medium used for respiration measurements consisted of 25 mmol/l KCL, 12.5 mmol/l morpholinopropane sulfonic acid, 1 mmol/l ethylene glycol-bis N,N,N',N'-tetra-acetic acid and 5 mmol/l potassium phosphate buffer (pH 7.4). The medium was equilibrated for 30 to 40 minutes with air in the oxygraph chambers and stirred at 750 rpm until a stable signal was obtained for calibration at air saturation. The corresponding oxygen concentration was calculated from the digitally recorded barometric pressure and the oxygen solubility at 37°C. The amplified signal was recorded in a computer with online display of the calibrated oxygen concentration and oxygen flux (negative time derivative of oxygen concentration; DatLab software for data acquisition and analysis; OROBOROS). Oxygen consumption was expressed as pmol/second per mg mitochondrial protein. Oxygen levels were always maintained above 40 nmol/ml.

Maximal oxidative capacities were determined in the presence of saturating concentrations of oxygen, ADP (0.25 mmol/l) and specific mitochondrial substrates. For complex I-dependent respiration, substrates were glutamate (10 mmol/l) plus malate (5 mmol/l), which provide nicotinamide adenine dinucleotide (NADH) to the respiratory chain (complex I activation). For measurement of complex II dependent respiration, first complex I was inhibited with rotenone (0.5 μmol/l), and then succinate (10 mmol/l) was added, which provides flavin adenine dinucleotide to the respiratory chain (complex II activation). The coupling of phosphorylation to oxidation was determined by calculating the respiratory control ratio (RCR) as the ratio between ADP-stimulated respiration (state 3) and respiration after ADP depletion (state 4).

### Calculations

The equations used I the present study are summarized in Table 1.

**Table 1:** Equations used in the present study

Parameter	Equation
Systemic oxygen delivery (ml/kg·minute)	Cardiac index (ml/kg·minute) × arterial oxygen content (ml/l)
Hepatosplanchnic oxygen delivery (ml/kg·minute)	Hepatic arterial blood flow (ml/kg·minute) + portal venous blood flow (ml/kg·min) × arterial oxygen content (ml/l)
Systemic oxygen consumption (ml/kg·min)	Cardiac index (ml/kg·min) × (arterial oxygen content [ml/l] – pulmonary artery oxygen content [ml/l])
Hepatic lactate uptake (μmol/kg·minute)	([Arterial lactate × hepatic arterial blood flow] + [portal vein lactate × portal vein blood flow]) – (hepatic vein lactate × [portal vein blood flow + hepatic arterial blood flow])
Hepatic lactate influx (μmol/kg·minute)	(Arterial lactate × hepatic arterial blood flow) + (portal vein lactate × portal vein blood flow)
Hepatic lactate extraction ratio	Hepatic lactate uptake/hepatic lactate influx

### Statistical analysis

The SPSS 13.0 software package (SPSS Inc., Chicago, IL, USA) was used for statistical analysis. Because of the small numbers of animals in each group, nonparametric test were used: Mann Whitney U-test for single measurements, and nonparametric analysis of variance for repeated measurements (Friedman test). In the latter case, differences between groups at certain time points were analyzed with the Mann Whitney U-test. For lactate and oxygen transport variables only baseline and end values were used. The two values were compared with the Wilcoxon test. Data are expressed as median and range. *P *< 0.05 was considered statistically significant.

## Results

The two groups received comparable total doses of endotoxin (7.3 [5.9 to 8.2] μg/kg in the control group and 7.9 [5.6 to 10.0] μg/kg in the norepinephrine group; *P *= 0.62) and total fluid input (5.9 [5.2 to 9.5] ml/kg per hour in the control group and 5.7 [5.1 to 7.5] ml/kg per hour in the norepinephrine group; *P *= 0.32).

### Systemic haemodynamics

**Figure 1:**
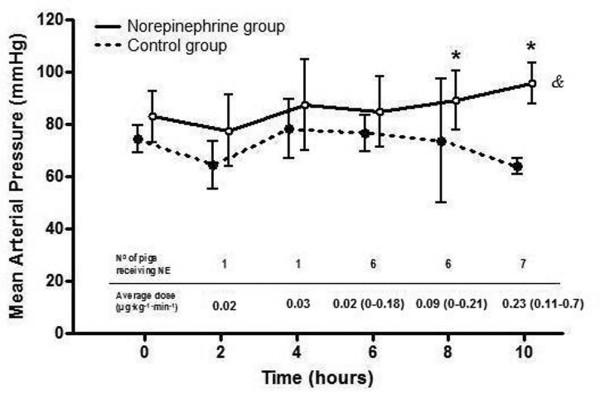
Evolution of MAP. Presented is the evolution of mean arterial pressure (MAP) during the experiment in the control group (black dotted line) and in the norepinephrine-treated group (black line). The table shows the number of pigs receiving norepinephrine and the average dose for each time point. Only the norepinephrine group exhibited a significant increase in MAP (^&^Friedman test; *P *= 0.019). Accordingly, values at 8 and 10 hours were higher in this group (*Mann Whitney U-test; *P *< 0.05 for both).

At baseline, before randomization, there were no significant differences between groups in terms of systemic haemodynamics and respiratory parameters (Table 2). After endotoxin administration, both groups exhibited a progressive increase in their mean pulmonary arterial pressures (*P *= 0.018 for controls and *P *= 0.003 for norepinephrine-treated animals) and in their pulmonary capillary wedge pressures (*P *= 0.016 for controls and *P *= 0.004 for norepinephrine-treated animals). Afterward, both parameters remained elevated until the end of the experiment, without differences between groups. Until hour 5 of the experiments, only one animal required norepinephrine in the norepinephrine group to reach the pressure goal specified in the protocol (Figure 1). From 6 to 8 hours, six animals required norepinephrine (average dose 0.09 [0.00 to 0.21] μg/kg per minute), and from 8 to 10 hours all animals needed norepinephrine to meet the specified goal (average dose 0.23 [0.11 to 0.70] μg/kg per minute; Figure 1).

**Table 2:** Time evolution of systemic hemodynamics and regional blood flows

Parameter		n	Baseline	2 hours	4 hours	6 hours	8 hours	10 hours	*P*^a^
Cardiac index (ml/minute per kg)	Control	6	99 (72–112)	104 (79–172)	71 (55–103)	72 (51–167)	88 (82–232)	117 (110–232)	0.002
	NE	7	107 (84–132)	96 (72–150)	70 (57–195)	103 (52–157)	100 (88–190)	161 (147–340)^b^	0.001
Heart rate (beats/min)	Control	6	122 (84–134)	120 (102–200)	126 (98–171)	124 (95–187)	122 (94–176)	128 (96–176)	0.7
	NE	7	105 (89–139)	122 (92–174)	120 (102–197)	123 (103–171)	157 (113–185)	202 (170–223)^b^	0.006
SVI (cardiac index/heart rate)	Control	6	0.77 (0.6–1.3)	0.88 (0.7–0.9)	0.57 (0.4–0.7)	0.58 (0.4–0.8)	0.81 (0.6–1.3)	0.94 (0.8–1.3)	0.003
	NE	7	0.95 (0.8–1.3)	0.86 (0.5–1.1)	0.66 (0.5–1)	0.86 (0.4–0.9)	0.81 (0.6–1.1)	0.76 (0.7–1.7)	0.045
MPAP (mmHg)	Control	6	12.5 (11–14)	25.5 (16–34)	29.5 (24–42)	29 (22–37)	30.5 (19–36)	25.5 (21–36)	0.018
	NE	7	12 (9–18)	29 (18–41)	30 (24–38)	24 (21–36)	30 (19–56)	25 (19–49)	0.003
PCWP (mmHg)	Control	6	2 (1–3)	6 (4–6)	5 (4–8)	4 (3–6)	5 (3–12)	3 (3–5)	0.016
	NE	7	3 (0–7)	7 (3–11)	6 (4–9)	5 (4–7)	6 (4–9)	5 (2–7)	0.004
Oxygenation index (mmHg/%)	Control	6	475 (445–512)	432 (354–496)	380 (316–507)	372 (313–413)	369 (272–410)	349 (326–398)	0.002
	NE	7	488 (407–548)	420 (367–445)	388 (294–453)	382 (317–496)	353 (195–478)	325 (222–390)	0.001
Hepatic artery flow (ml/minute per kg)	Control	5	3.7 (1.7–11.5)	2.6 (2–7.6)	1.1 (0.3–1.9)	2.1 (0.5–2.9)	4.5 (0.6–5.5)	3.5 (1.7–5.8)	0.037
	NE	7	3.9 (0.5–7.9)	1.4 (0.9–6.5)	1.3 (0.1–2.7)	1.2 (0.2–7.2)	2.8 (0.6–7.3)	3 (0.3–8.9)	0.002
Portal vein flow (ml/minute per kg)	Control	6	20 (16–22)	16.9 (12–23)	11.5 (6–14)	11.9 (9–16)	16.2 (10–32)	18.6 (13–32)	<0.001
	NE	7	19.6 (12–26)	13.8 (10–20)	11.6 (8–16)	14 (10–22)	15.8 (12–27)	19.1 (12–31)	0.001

Data are presented as median (range). ^a^Friedman test for each group and variable in time. ^b^Mann Whitney U-test between groups at the corresponding time point. MPAP, mean pulmonary artery pressure; NE, norepinephrine group; oxygenation index, arterial oxygen tension/fractional of inspired oxygen; PCWP, pulmonary capillary wedge pressure; SVI, stroke volume index.

As a result of the intervention, there was a significant increase in MAP only in the norepinephrine-treated animals (in controls: from 73 [69 to 81] to 63 [60 to 68] mmHg [*P *= 0.09]; in norepinephrine-treated animals: from 83 [69 to 93] to 96 [86 to 108] mmHg [*P *= 0.019]). Accordingly, MAP levels at 8 and 10 hours were higher than in controls (*P *< 0.05 for both time points; Figure 1). Cardiac index increased in both groups (*P *< 0.003 for both groups), but end values were significantly higher in the norepinephrine-treated group (41% in controls versus 81% in norepinephrine-treated animals; *P *= 0.022). The observed increase in cardiac index was mainly the result of an increase in the heart rate only in the norepinephrine-treated animals (*P *= 0.006; Table 2).

### Regional blood flows

**Figure 2:**
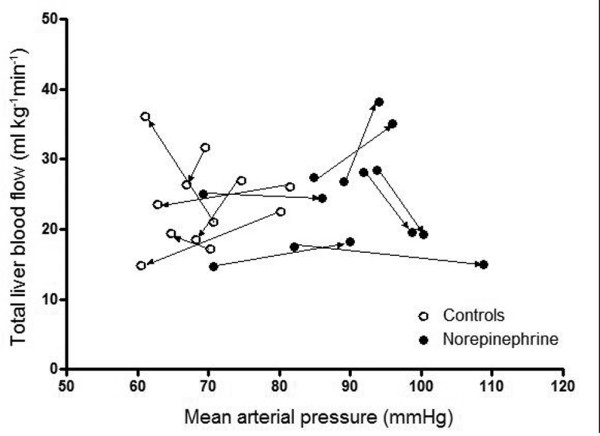
Relation between MAP and liver total flow. Each line shows the evolution for each pig during the experiment, from baseline to end values. No consistent relationship between changes in mean arterial pressure (MAP) and changes in liver total blood flow was observed in either group.

The two groups exhibited a similar decrease in hepatic and portal flows until 4 to 6 hours after endotoxin infusion; both hepatic and portal flows recovered to baseline values at the end of the experiment in both groups (P < 0.002 for both vessels; Table 2). There was no consistent relationship between changes in MAP and changes in liver total blood flow in both groups (Figure 2).

### Oxygen delivery and consumption

Systemic oxygen delivery significantly increased from baseline to the end of the experiment in both groups (*P *< 0.03 for both groups), but end values were significantly higher in the norepinephrine-treated animals than in the control group (*P *= 0.001). Systemic oxygen consumption increased in both groups (*P *< 0.05 for both groups) but with no differences between them. Accordingly, systemic oxygen extraction decreased over time only in the norepinephrine-treated group (*P *= 0.013). Hepatosplanchnic oxygen delivery and consumption did not change over time and were not different between groups (Table 3).

**Table 3:** Time evolution of oxygen transport variables

Parameter		n	Baseline	End	*P*^a^
DO_2 _systemic (ml/minute per kg)	Control	6	12.8 (11–15)	16.3 (15–24)	0.028
	NE	7	16.4 (12–19)	29.3 (25–52)^b^	0.018
VO_2 _systemic (ml/minute per kg)	Control	5	4.5 (3–5)	5.7 (4–11)	0.043
	NE	7	5.2 (3–7)	6.9 (6–10)	0.028
DO_2 _hepatosplanchnic (ml/minute per kg)	Control	6	3.1 (2.6–4.1)	3.2 (1.8–3.9)	0.46
	NE	7	3.7 (2.3–4.2)	3.3 (2.7–5.7)	0.4
VO_2 _hepatosplanchnic (ml/minute per kg)	Control	6	1.5 (0.6–2.1)	1.8 (0.6–2.2)	0.46
	NE	7	1.2 (0.8–1.7)	1.4 (0.5–2.1)	0.4

Data are presented as median (range). ^a^Wilcoxon test for each group and variable in time. ^b^Mann Whitney U-test between groups at the corresponding time point. NE, norepinephrine; DO_2_, oxygen delivery; VO_2_, oxygen consumption.

### Lactate handling

For lactate exchange calculations, in three pigs (two control pigs and one norepinephrine-treated animal) the portal vein was not sampled. Lactate values were similar in all measured vessels at baseline (Table 4). Afterward, arterial and hepatic vein lactate levels increased over time in both groups (*P *= 0.028 for both vessels; Table 4). Norepinephrine-treated animals exhibited a significant increase in the hepatic lactate influx (*P *= 0.028), whereas the hepatic lactate uptake exhibited a tendency to decrease only in the control group (*P *= 0.07). Accordingly, the hepatic lactate extraction ratio decreased over time in both groups (Table 4).

**Table 4:** Time evolution of lactate concentrations and hepatic lactate exchange

Parameter		n	Baseline	End	*P*^a^
Arterial lactate (mmol/l)	Control	6	0.67 (0.3–0.7)	0.92 (0.6–1.1)	0.028
	NE	7	0.66 (0.4–0.8)	1.1 (0.7–2.1)	0.028
Hepatic vein lactate (mmol/l)	Control	6	0.45 (0.4–0.6)	0.71 (0.5–1.1)	0.028
	NE	6	0.41 (0.3–0.5)	0.89 (0.7–1.7)	0.028
Portal vein lactate (mmol/l)	Control	4	0.74 (0.6–0.9)	1 (0.6–1.23)	0.14
	NE	7	0.68 (0.5–0.9)	1.1 (0.9–1.9)	0.028
Hepatic lactate influx (μmol/minute per kg)	Control	4	18.1 (15–21)	21.8 (15–24)	0.3
	NE	6	18.3 (8–24)	38.5 (14–42)	0.028
Hepatic lactate uptake (μmol/minute per kg)	Control	4	7.4 (7–12)	4.1 (0.3–10)	0.07
	NE	6	6.6 (4–11)	5.7 1.7–13)	0.6
Hepatic lactate extraction ratio (%)	Control	4	44 (38–60)	22 (2–41)	0.05
	NE	6	40 (29–51)	17 (9–34)	0.046

Data are presented as median (range). ^a^Wilcoxon test for each group and variable in time. NE, norepinephrine.

### Liver mitochondrial function

**Figure 3:**
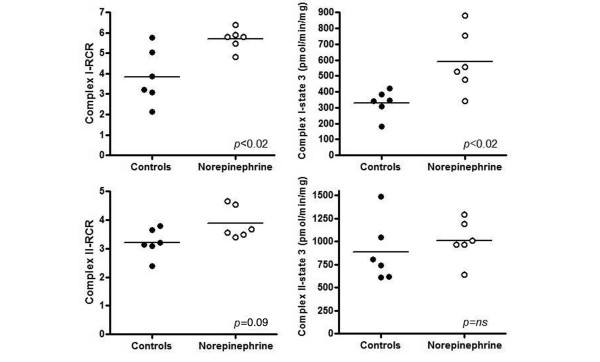
Complex I-dependent and complex II-dependent liver mitochondrial respiration. State 3: equivalent to maximal mitochondrial respiration. *P *values from unpaired *t*-test comparison between groups. Data obtained by high-resolution Oxygraph (Oroboros, DatLab software for data acquisition and analysis, Graz, Austria).

At the end of the experiment, norepinephrine-treated animals exhibited higher levels of liver mitochondria respiration than did control animals, as indicated by higher values of their RCR for complex I (3.5 [2.1 to 5.7] for controls animals versus 5.8 [4.8 to 6.4] for norepinephrine-treated animals; *P *= 0.015) and a tendency toward higher RCR for complex II (3.1 [2.3 to 3.8] for controls versus 3.7 [3.3 to 4.6] for norepinephrine-treated animals; *P *= 0.09). Also, the maximal liver mitochondrial respiration (state 3) for complex I was significantly higher in the norepinephrine-treated group (343 [181 to 422] pmol/second per mg for controls versus 539 [340 to 879] pmol/second per mg for norepinephrine-treated animals; *P *= 0.026; Figure 3). Resting respiration (state 4; complex I: 86 [60 to 138] pmol/second per mg for controls versus 96 [62 to 151] pmol/second per mg for norepinephrine-treated animals [*P *= 0.31]; and complex II: 231 [193 to 623] pmol/second per mg for controls versus 276 [174 to 283] pmol/second per mg for norepinephrine-treated animals [*P *= 0.39]; Figure 3) and citrate synthase activity (12.6 [11.1 to 17.0] versus 16.6 [11.3 to 20.5], respectively [*P *= 0.3]) were similar between groups. Previous results in similar control and endotoxin-treated animals are compared with the current results in Table 5.

**Table 5:** Liver complex I-dependent mitochondrial respiration

	Previous controls^a^	Previous septic^a^	Current septic	Current septic + NE
State 3	343 (324–371)	247 (204–296)	343 (181–422)	539 (340–879)
State 4	61 (55–74)	90 (74–101)	86 (60–138)	96 (62–151)
RCR (state 3/state 4)	5.6 (5–6)	2.8 (2.4–3.2)	3.5 (2.1–5.7)	5.8 (4.8–6.4)

Shown are is a comparison of liver complex I-dependent mitochondrial respiration with previous results from similarly instrumented animals with and without endotoxin exposure. Data are presented as median (range). ^a^Data from previous study [29]: pigs were randomized for 12 hours to control or endotoxaemia (0.4 μg/kg per hour) and were instrumented similar to the present study.

## Discussion

In our model, endotoxaemia was associated with increasing levels of cardiac index and mean pulmonary artery pressure, whereas addition of norepinephrine was associated with higher levels of MAP and further increases in cardiac index, heart rate and systemic oxygen delivery. Despite this, norepinephrine treatment was not associated with changes in stroke volume, total liver blood flow, or hepatosplanchnic oxygen consumption, or with an improvement in the hepatic lactate exchange. Surprisingly, with unaltered hepatic oxygen transport, the animals treated with norepinephrine exhibited an increase in efficiency of their liver mitochondrial respiration when compared with septic animals not treated with norepinephrine.

The haemodynamic and metabolic effects of norepinephrine during sepsis are controversial, several studies have been performed to provide insight. LeDoux and coworkers [22] studied 10 patients with septic shock and found that increasing MAP from 65 to 85 mmHg with norepinephrine was related to significant increases in cardiac index, left ventricular stroke work index, heart rate and systemic oxygen delivery. However, it was not associated with changes in systemic oxygen consumption, lactate concentrations, capillary blood flow, urine output, or gastric mucosal partial carbon dioxide tension. This suggests that higher blood pressure levels are not associated with better organ perfusion during sepsis. Similarly, our data reveal no consistent relationship between MAP levels and liver blood flow, and norepinephrine was not associated with a greater increase in systemic oxygen consumption in comparison with septic animals not receiving norepinephrine.

Other studies, however, have identified some beneficial effects of norepinephrine during sepsis. For example, Treggiari and coworkers [20], in an animal model, showed that increasing MAP with norepinephrine by 10 mmHg above the baseline level of 50 mmHg was associated with an increase in portal vein flow and almost restoration of renal and mucosal flows to pre-shock levels. Further increases in MAP were not associated with more benefit. Also, one crossover study [16] conducted in septic patients compared replacement of dopamine with norepinephrine to achieve a MAP goal of 80 mmHg. This study revealed that the drugs were associated with similar splanchnic blood flow and hepatic oxygen consumption, but that patients treated with norepinephrine exhibited higher levels of hepatic lactate uptake. This suggests that norepinephrine may improve liver metabolic function during sepsis independent of its regional haemodynamic effects. This is in contrast to our findings in septic pigs, in which norepinephrine was associated with an increase in the hepatic lactate influx with no concomitant increase in the hepatic lactate uptake, suggesting that the capacity of the liver to increase the lactate uptake was exhausted and not improved by norepinephrine.

Our study shows that both complex I and II respiratory efficiency was increased by the use of norepinephrine during endotoxaemia in pigs. Septic pigs not treated with norepinephrine exhibited decreased RCR (an index of respiratory efficiency), similar to those in septic pigs from previous studies [24,29,30] (Table 5). In the present study the addition of norepinephrine was associated with liver mitochondria RCRs for complexes I and II similar to those reproted previously by us and others in nonseptic control pigs, suggesting that norepinephrine may restore respiratory efficiency (Table 5). Respiratory control depends on the presence of a chemiosmotic gradient generated by the coupled passage of protons from the mitochondrial matrix to the mitochondrial inter-membrane space during the electron flux through the electron transport chain. The presence and magnitude of this chemiosmotic gradient regulates and limits the flux of electrons through the electron transport chain, and secondarily mitochondrial oxygen consumption. In our study, both groups exhibited similar increased values for their state 4 respiration (resting respiration, which is mitochondrial oxygen consumption by isolated mitochondria induced by a particular substrate, in the absence of ADP) [29]. This result confirms that during endotoxaemia the mitochondrial membrane is damaged, with a secondary increase in the loss of protons back to the matrix or to the cytoplasmatic space, which is not coupled with ATP production. This reduction in the chemiosmotic gradient is coupled with an increase in the electron flux and the oxygen consumption, which was not prevented by norepinephrine (Table 5).

The observed improvement in both complex I-dependent and complex II-dependent RCRs in septic animals treated with norepinephrine, into the normal range, in spite of their increased resting respiration (decreased chemiosmotic gradient), was mainly due to an increase in their mitochondrial state 3 respiration (maximal respiration, which is mitochondrial oxygen consumption under saturating ADP concentrations stimulated by a particular combination of substrates). Norepinephrine-treated septic pigs exhibited complex I-dependent state 3 respiration values that were higher than those of septic pigs that did not receive norepinephrine, and higher than those of control pigs from our previous experiments [29] (Table 5). This suggests that norepinephrine, by some mechanism, improves the rate of oxygen consumption in the presence of an excess amount of ADP. The RCR is considered to indicate the degree of coupling, especially when basal state 4 respiration changes at different conditions. In our study, state 4 respiration was not different between groups, indicating that proton leak was unaffected by the different treatment. Therefore, state 3 respiration and RCR represent almost exactly the same phenomena, and no further information is gained by the concomitant change in RCR.

Previous studies conducted in isolated or perfused livers from nonseptic small animals have shown that norepinephrine increases cellular respiration in a dose-dependent manner, and that this effect is blocked by the addition of an α-antagonist (for example, phenoxybenzamine), but not by the addition of a β-antagonist (for example, propanolol). It has also been shown that the increase in cellular respiration depends on the extracellular calcium concentration [27]. Even more, exposure of isolated hepatocytes to physiological concentrations of norepinephrine is related to an increase in cytosolic calcium levels and to an active transport of calcium into the mitochondrial matrix [31]. In liver cells, calcium can stimulate three different dehydrogenases of the citrate cycle, increasing the substrate availability of NADH to the respiratory chain and thus increasing mitochondrial respiration [26]. However, it has also been suggested that an increase in mitochondrial oxygen consumption associated with the increase in calcium concentrations may be related to an increase in mitochondrial membrane potential and a secondary increase in reactive oxygen species production [32,33].

A limitation of our study was the normotensive haemodynamic situation of the groups, although in many ICUs norepinephrine would be used in this clinical situation. A further limitation is the lack of assessment of the liver microcirculation, which can be influenced by norepinephrine, improving cellular oxygen delivery, although mitochondrial dysfunction may occur even in the absence of tissue hypoxia [23,34]. A third imitation is the lack of liver ATP levels, which should be addressed in future studies. A final limitation is the fact that it is difficult to establish whether the observed changes in liver mitochondrial respiration are a result of the increased blood pressure or a direct effect of the compound norephinephrine. In our opinion, a direct effect of blood pressure is unlikely; rather, an effect of concomitantly increased oxygen delivery may be expected. However, neither hepatic oxygen delivery nor consumption was altered by the addition of norepinephrine. We recently showed that adding more volume in our model of sepsis leads to similar pressures [35] and that mitochondrial function is also impaired by volume [36].

## Conclusion

Our study shows that norepinephrine treatment, using clinically relevant doses that are commonly applied in patients with sepsis [11,37], during endotoxaemia to control blood pressure improves liver mitochondria complex I-dependent and complex II-dependent efficiency of respiration. This effect was mainly explained by an increase in liver mitochondria maximal respiration and was probably mediated by a direct effect of norepinephrine on liver cells.

## Key messages

• Endotoxaemia was associated with increased levels of cardiac index and mean pulmonary arterial pressure, whereas the addition of norepinephrine was associated with higher levels of MAP and further increases in cardiac index and systemic oxygen delivery.

• Norepinephrine treatment was not associated with changes in the stroke volume, in hepatosplanchnic oxygen consumption, or an improvement in the hepatic lactate exchange.

• No consistent relationship between changes in MAP and changes in liver total blood flow were observed.

• Norepinephrine-treated animals exhibited increased efficiency in liver mitochondrial respiration when compared with septic animals with no norepinephrine.

• The improvement in liver mitochondrial respiration was mainly accounted for by an increase in their maximal respiration and was probably mediated by a direct effect of norepinephrine on liver cells.

## Abbreviations

MAP = mean arterial pressure; NADH = nicotinamide adenine dinucleotide; RCR = respiratory control ratio.

## Competing interests

The authors declare that they have no competing interests.

## Authors' contributions

SMJ and JT devised the study protocol. TR, BB, SMJ, SB, SD and JG initiated and performed all animal experiments. TR, SD and PL performed mitochondria-related experiments. TR and SMJ analyzed the data. All the authors contributed to and approved the final manuscript.
